# Altered structural hippocampal intra-networks in a general elderly Japanese population with mild cognitive impairment

**DOI:** 10.1038/s41598-023-39569-6

**Published:** 2023-08-16

**Authors:** Sera Kasai, Keita Watanabe, Yoshihito Umemura, Yuka Ishimoto, Miho Sasaki, Haruka Nagaya, Soichiro Tatsuo, Tatsuya Mikami, Yoshinori Tamada, Satoru Ide, Masahiko Tomiyama, Masashi Matsuzaka, Shingo Kakeda

**Affiliations:** 1 Department of Radiology, Graduate School of Medicine, Hirosaki University, Hirosaki, Japan; 2 Department of Radiology, Kyoto Prefectural University of Medicine, 465 Kajiimachi, Jokyo-ku, Kyoto-shi, Kyoto-fu Japan; 3 Innovation Center for Health Promotion, Hirosaki University, Hirosaki, Japan; 4 Department of Radiology, University of Occupational and Environmental Health, School of Medicine, Kitakyushu, Japan; 5 Department of Neurology, Graduate School of Medicine, Hirosaki University, Hirosaki, Japan; 6 Department of Medical Informatics, Hirosaki University Hospital, Hirosaki, Japan

**Keywords:** Neurological disorders, Neurological disorders

## Abstract

Although altered networks inside the hippocampus (hippocampal intra-networks) have been observed in dementia, the evaluation of hippocampal intra-networks using magnetic resonance imaging (MRI) is challenging. We employed conventional structural imaging and incident component analysis (ICA) to investigate the structural covariance of the hippocampal intra-networks. We aimed to assess altered hippocampal intra-networks in patients with mild cognitive impairment (MCI). A cross-sectional study of 2122 participants with 3T MRI (median age 69 years, 60.9% female) were divided into 218 patients with MCI and 1904 cognitively normal older adults (CNOA). By employing 3D T1-weighted imaging, voxels within the hippocampus were entered into the ICA analysis to extract the structural covariance intra-networks within the hippocampus. The ICA extracted 16 intra-networks from the hippocampal structural images, which were divided into two bilateral networks and 14 ipsilateral networks. Of the 16 intra-networks, two (one bilateral network and one ipsilateral networks) were significant predictors of MCI from the CNOA after adjusting for age, sex, education, disease history, and hippocampal volume/total intracranial volume ratio. In conclusion, we found that the relationship between hippocampal intra-networks and MCI was independent from the hippocampal volume. Our results suggest that altered hippocampal intra-networks may reflect a different pathology in MCI from that of brain atrophy.

## Introduction

Cognitive impairment in the elderly population is a growing concern worldwide. Mild cognitive impairment (MCI) is characterized by decline in cognitive functions such as memory and attention, which is the prodromal stage of Alzheimer’s disease (AD). Early intervention for dementia is essential to slow the progression to AD. Therefore, the identification of MCI has been a sustained research focus for decades. The hippocampus plays a key role in cognitive processes and is composed of different subfields, including the four cornu ammonis fields (CA1–4), dentate gyrus (DG), and subiculum. Hippocampal volume, as measured by structural magnetic resonance imaging (sMRI), may serve as an indication of atrophy and neurodegeneration associated with AD or MCI^1^, despite the presence of interindividual variability in hippocampal volume among aged subjects^2^. The hippocampus is a complex structure where a region with functionally and structurally distinct subfields. Because the hippocampal volume loss is associated with AD pathology, hippocampal subfield volume measurement may represent a more sensitive indicator of AD development than total hippocampal volume measurement. Therefore, it may be important to investigate the role of hippocampal subfields in patients with cognitive impairment.

From the perspective of brain networks, the hippocampus is considered to be a hub region for multiple cognitive processes^3^. Using resting-state functional MRI (rs-fMRI), many previous studies have shown functional connectivity disruption between the hippocampus and other brain regions such as the posterior cingulate, medial prefrontal, inferior parietal, and lateral temporal cortex in patients with AD and MCI^4^. Recently, some studies have used hippocampal subfields as seed regions in rs-fMRI analyses. A rs-fMRI study with healthy participants showed that several hippocampal subregions (CA1, dentate gyrus, and subiculum) emerged as functional network hubs^5^. Flores et al. also assessed connectivity specificities of hippocampal subfields and their changes in early AD using rs-fMRI and found that amnestic type MCI patients showed reduced connectivity within the subiculum network (defined as the subiculum connected with frontal and posterior cingulate regions)^6^. Although these studies evaluated the specificity of hippocampal subfield intrinsic connectivity when compared to the rest of the brain, few studies have assessed the specificity of hippocampal subfield intrinsic connectivity^7,8^. Actually, it has been known that a complex network exists in the hippocampus^9^. Ezama et al. showed an activity hotspot among hippocampal subfields in 172 healthy participants with high spatial-resolution rs-fMRI at 7T and found that two networks (CA1, CA3, CA4, and dentate gyrus fields and subiculum, CA4, and dentate gyrus fields) were subserved by co-activity. Dalton et al. also showed subfield-to-subfield functional connectivity within the hippocampus in healthy participants. However, to our knowledge, no study has evaluated the intrinsic connectivity of the hippocampal subfield in populations with MCI.

Recently, a source-based morphometry (SBM) technique using 3D T1-weighted imaging (T1WI) data has been introduced This data-driven multivariate approach can identify patterns across different voxels without prior anatomical knowledge^10,11^. SBM applies an independent component analysis (ICA), arranges voxels into groups that contain similar information^10^, and acquires common morphological features which represent brain networks among individuals. Structural imaging has the advantage of high spatial resolution, which is considered important for evaluating small regions like the hippocampus. Moreover, this method is suitable for identifying novel networks and may be a useful tool for brain MRI studies with larger populations. More recently, Watanabe et al. reported that a detailed intra-network in the hippocampus (hippocampal intra-network) could be extracted using this method^12^. Thus, we reviewed data from a population-based prospective study of cerebro- and cardiovascular diseases and dementia in a large population of older Japanese individuals (the Iki-Iki study) and acquired hippocampal intra-networks using SBM from the MRI data. To our knowledge, there have been no population-based studies with large sample sizes to assess the association between MCI status and hippocampal intra-network connectivity. Our aim was to assess whether individuals with MCI have altered hippocampal intra-network connectivity as measured using SBM when compared with cognitively normal older adults (CNOA).

## Results

### Demographic data

For the clinical characteristics between the included excluded participants, there were no significant differences in age, sex, years of education, self-reported medical history (hypertension, hyperlipidemia, or diabetes), and diagnosis group (MCI and CNOA) (see Supplementary Table S1 online). Table 1 presents participant demographic data, which was included in the analyses. There were significant differences in age, sex, years of education, and dyslipidemia between individuals with MCI and CNOA; however, there were no significant differences in diabetes mellitus or hypertension status.

**Table 1:** Clinical characteristics of 2122 participants.

	Total (n = 2122)	MCI (n = 218)	NOA (n = 1904)	p value
Age, median (IQR)	69 (67–73)	72 (69–76)	69 (66–73)	< 0.001
Sex: male/female	823/1299	120/98	703/1201	< 0.001
Diabetes, n (%)	271 (12.8%)	33 (15.1%)	238 (12.5%)	0.149
Hypertension, n (%)	980 (46.2%)	106 (48.6%)	874 (65.9%)	0.268
Hyperlipidemia, n (%)	926 (43.6%)	77 (35.3%)	849 (44.6%)	0.023
Education: University/High school/Junior high school	623/1108/391	51/84/83	572/1024/308	< 0.001
GMV/ICV ratio, median (IQR)	0.42 (0.40–0.44)	0.41 (0.39–0.42)	0.42 (0.40–0.44)	< 0.001
WMV/ICV ratio, median (IQR)	0.33 (0.31–0.34)	0.32 (0.30–0.33)	0.33 (0.31–0.34)	< 0.001
HV/ICV ratio, median (IQR)	0.0027 (0.0028–0.003)	0.0027 (0.0025–0.0029)	0.0028 (0.0027–0.003)	< 0.001

*MCI* mild cognitive impairment, *NOA* normal older adult, *IQR* interquartile range, *ICV* total intracranial volume, *GMV* total gray matter volume, *WMV* total white matter volume, *HV* hippocampal volume.

Compared to the CNOA group, the participants with MCI had significantly reduced total GM volume (GMV)/intracranial volume (ICV), total white matter volume (WMV)/ICV, and hippocampal volume (HV)/ICV ratios.

### Intra-network in the hippocampus

**Figure 1:**
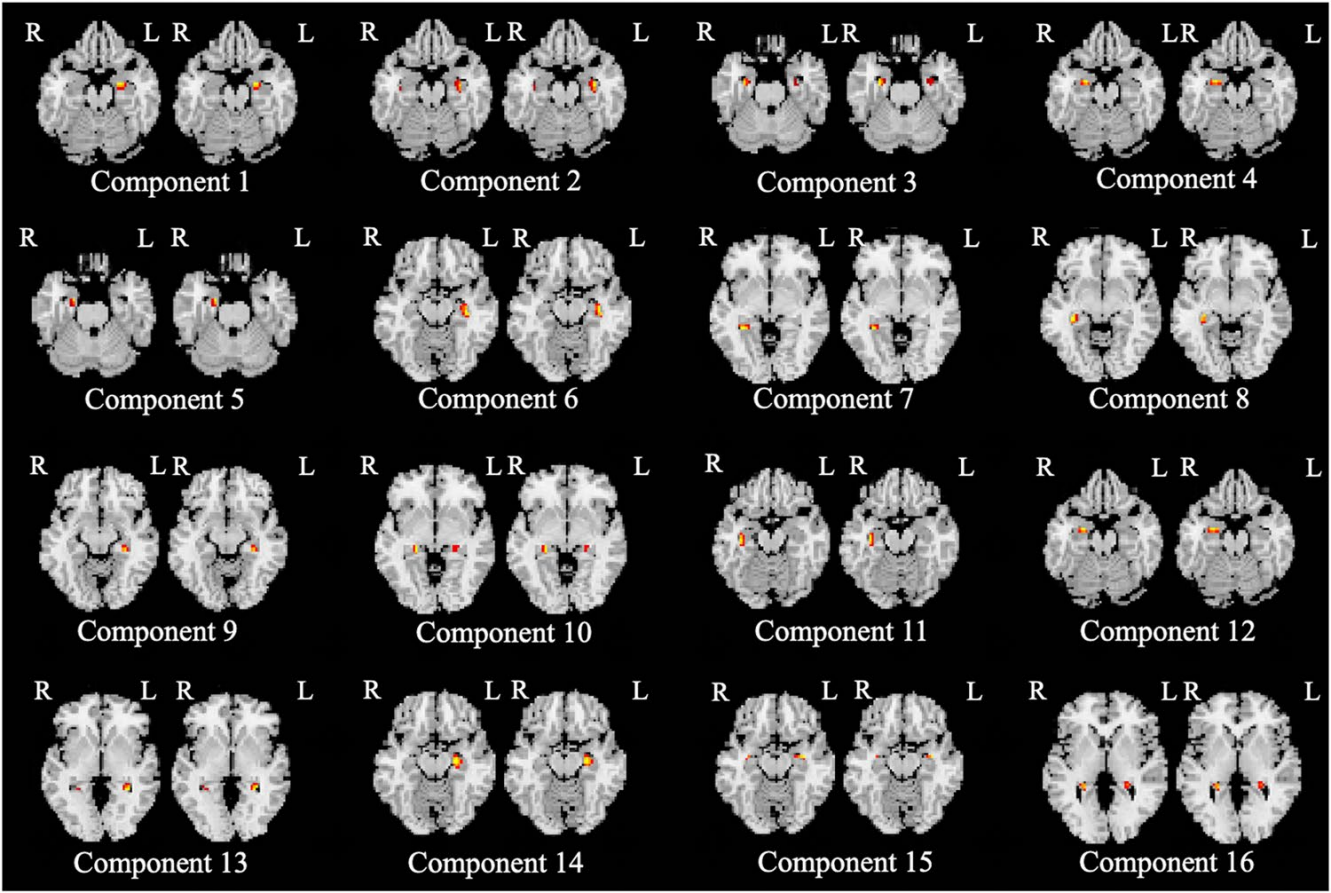
Voxel-based structural covariance intra-network in the hippocampus. The figure shows the structural covariance networks with |Z|> 2.5. Independent component analysis generated 16 independent components (Component 1–16). Two networks, including the right hippocampal head (Component 5) and bilateral hippocampal tail (Component 16) were significant independent predictors of MCI. Component 5: Right hippocampal head (CA1), which mainly consists of a portion of CA1. Component 16: Bilateral hippocampal tail, mainly consisting of a portion of CA1.

Independent component analysis (ICA) generated 16 independent components (Fig. 1). The components were reviewed by two experienced neuroradiologists. The hippocampal subfield regions of independent components were determined with the consent of neuroradiologists based on an illustrated tutorial research paper^13^. No components were determined to be artifacts using the criteria by Xu et al.^10^. Table 2 shows the independent components representing structural covariance networks in the hippocampus.

**Table 2:** An independent predictor of MCI.

	Model 1		Model 2		Model 3		Model 4		Model 5	
	OR (95% CI)	p value	OR (95% CI)	p value	OR (95% CI)	p value	OR (95% CI)	p value	OR (95% CI)	p value
Female sex	0.55 (0.39, 0.78)	< 0.001	0.48 (0.36, 0.65)	< 0.001	0.57 (0.42, 0.79)	< 0.001	0.40 (0.28, 0.57)	< 0.001	0.44 (0.29, 0.67)	< 0.001
Age (per year)	1.11 (1.07, 1.15)	< 0.001	1.11 (1.07, 1.15)	< 0.001	1.09 (1.05, 1.13)	< 0.001	1.09 (1.04, 1.13)	< 0.001	1.08 (1.04, 1.13)	< 0.001
Hypertension, n (%)	1 (1, 1)	0.33	1 (1, 1)	0.32	1 (1, 1)	0.33	1 (1, 1)	0.35	1 (1, 1)	0.36
Hyperlipidemia, n (%)	0.78 (0.58, 1.06)	0.13	0.77 (0.57, 1.05)	0.095	0.79 (0.58, 1.07)	0.13	0.77(0.56, 1.05)	0.10	0.78(0.57, 1.06)	0.12
Diabetes, n (%)	1 (1, 1)	0.0039	1 (1, 1)	0.0041	1 (1, 1)	0.04	1 (1, 1)	0.08	1 (1, 1)	0.08
Education (high school)	0.89 (0.61, 1.29)	0.53	0.88 (0.61, 1.28)	0.51	0.9 (0.62, 1.3)	0.56	0.88 (0.60, 1.28)	0.50	0.89 (0.61, 1.3)	0.54
Education (junior high school)	2.57 (1.73, 3.81)	< 0.001	2.53 (1.71, 3.74)	< .001	2.62 (1.76, 3.88)	< 0.001	2.47 (1.65, 3.70)	< 0.001	2.51 (1.67, 3.76)	< 0.001
HV/ICV					0.76 (0.65, 0.9)	0.001			0.87 (0.67, 1.14)	0.31
GMV/ICV	0.86 (0.72, 1.03)	0.11								
WMV/ICV			0.93 (0.80, 1.1)	0.40						
Component 1							0.84 (0.68, 1.03)	0.09	0.85 (0.69, 1.04)	0.12
Component 2							1.10 (0.90, 1.35)	0.33	1.12 (0.92, 1.37)	0.27
Component 3							0.96 (0.77, 1.21)	0.75	0.95 (0.75, 1.19)	0.63
Component 4							0.88 (0.71, 1.09)	0.23	0.91 (0.73, 1.13)	0.37
Component 5							0.77 (0.62, 0.96)	0.021	0.77 (0.62, 0.96)	< 0.001
Component 6							0.73 (0.58, 0.91)	0.005	0.74 (0.59, 0.92)	0.01
Component 7							1.01 (0.82, 1.26)	0.89	1.02 (0.82, 1.26)	0.88
Component 8							1.02 (0.77, 1.34)	0.90	1.05 (0.79, 1.39)	0.75
Component 9							0.96 (0.78, 1.18)	0.72	0.95 (0.78, 1.17)	0.65
Component 10							1.08 (0.87, 1.33)	0.50	1.07 (0.87, 1.32)	0.52
Component 11							0.80 (0.66, 0.98)	0.031	0.81 (0.66, 0.99)	0.038
Component 12							0.99 (0.78, 1.27)	0.95	1.02 (0.8, 1.32)	0.85
Component 13							1.05 (0.83, 1.32)	0.71	1.04 (0.82, 1.31)	0.76
Component 14							0.98 (0.80, 1.21)	0.88	1.01 (0.81, 1.25)	0.96
Component 15							1.16 (0.93, 1.44)	0.18	1.1 (0.86, 1.4)	0.45
Component 16							0.66 (0.54, 0.82)	< 0.001	0.66 (0.53, 0.82)	< 0.001

*MCI* mild cognitive impairment, *ICV* total intracranial volume, *GMV* total gray matter volume, *WMV* total white matter volume, *HV* hippocampal volume, *CI* confidence interval, *OR* odds ratio.

Bold indicates statistically significant after Bonferroni correction.

### Intra-network alternation in the hippocampus

The HV/ICV ratio was a significant independent predictor of MCI (P = 0.0012) after adjustment (Model 3), whereas the GMV/ICV ratio (Model 1, P = 0.11) and WMV/ICV ratio (Model 2, P = 0.40) were not significant independent predictor of MCI (Table 2). After adjustment for the HV/ICV ratio in addition to the potential confounders, two networks, including the right hippocampal head (Component 5) and bilateral hippocampal tail (Component 16) were significant independent predictors of MCI (p < 0.001 and < 0.001, respectively) (Model 5). Importantly, when HV/ICV was added to the model (Model 5), these two networks were independent predictors of MCI, whereas HV/ICV (p = 0.31) was not.

Each logistic regression model was subjected to the Hosmer–Lemeshow test that assesses the fit of the model under the null hypothesis that "the model is good". In results, Model 1,3, 4, and 5 was defined as good (Model 1: p-value = 0.543, χ^2^ = 6.941; Model 3: p-value = 0.41, χ^2^ = 8.248; Model 4: p-value = 0.051, χ^2^ = 15.452; Model 5: p-value = 0.158, χ^2^ = 11.851), which indicate the null hypothesis is not rejected. Alternatively, Model 2 rejected the null hypothesis (p-value = 0.0157, χ^2^ = 18.856)^14^.

For the Model 5, variance inflation factor (VIF) values were calculated to measure the possible collinearity between the HV/ICV ratio and the 16 independent components. The results indicated no collinearity between them (mean VIF = 1.47, max VIF = 1.95 and minimum = 1.00), because a VIF of > 5 indicates high correlation of the variables^15^. Pearson’s correlation coefficients were also calculated to detect collinearity among them. Although there were significant correlations between HV/ICV ratio and 16 independent components, a correlation coefficient of < 0.5 between them (Table 3), which was considered indicative of no multicollinearity^16^.

**Table 3:** Relationship (correlation coefficients) among hippocampal volume and 16 independent components.

	HV	C1	C2	C3	C4	C5	C6	C7	C8	C9	C10	C11	C12	C13	C14	C15	C16
HV	–																
C1	− 0.01	–															
C2	− 0.16***	0.34***	–														
C3	− 0.31***	0.39***	0.36***	–													
C4	0.27***	0.31***	− 0.05*	0.30***	–												
C5	− 0.14***	0.37***	− 0.05**	0.48***	0.39***	–											
C6	0.28***	− 0.14***	− 0.10***	− 0.38***	− 0.17***	− 0.32***	–										
C7	− 0.11***	− 0.26***	− 0.12***	− 0.21***	− 0.36***	− 0.08***	− 0.23***	–									
C8	0.28***	− 0.51***	− 0.42***	− 0.59***	− 0.27***	− 0.41***	0.36***	0.24***	–								
C9	− 0.08***	− 0.22***	− 0.16***	− 0.24***	− 0.31***	− 0.04*	0.03	0.49***	0.19***	–							
C10	− 0.22***	− 0.34***	− 0.12***	− 0.20***	− 0.45***	− 0.23***	− 0.08***	0.55***	0.20***	0.45***	–						
C11	0.18***	− 0.38***	− 0.16***	− 0.19***	− 0.07**	− 0.18***	0.03	0.15***	0.32***	0.12***	0.07**	–					
C12	0.25***	− 0.35***	− 0.30***	− 0.49***	− 0.24***	− 0.33***	0.47***	0.21***	0.55***	0.40***	0.23***	− 0.04	–				
C13	− 0.40***	− 0.29***	0.10***	− 0.03	− 0.45***	− 0.26***	− 0.15***	0.34***	0.03	0.29***	0.40***	− 0.10***	0.21***	–			
C14	0.20***	0.37***	0.03	− 0.01	0.19***	0.31***	− 0.03	− 0.14***	− 0.33***	0.08***	− 0.26***	− 0.13***	− 0.11***	− 0.33***	–		
C15	− 0.45***	− 0.24***	0.12***	− 0.09***	− 0.47***	− 0.37***	0.13***	0.18***	0.15***	0.13***	0.35***	− 0.06**	0.17***	0.52***	− 0.48***	–	
C16	− 0.32***	− 0.31***	− 0.08**	− 0.21***	− 0.42***	− 0.30***	− 0.07***	0.35***	0.28***	0.21***	0.49***	− 0.20***	0.32***	0.51***	− 0.31***	0.43***	–

*HV* Hippocampal volume, *C* component.

*p < 0.05, **p < 0.01, ***p < 0.001.

The network of the right hippocampal head (Component 5) mainly consisted of a portion of CA1 and bilateral hippocampal tail (Component 16) of CA1 (Fig. 1).

## Discussion

In the present study, we assessed networks in the hippocampus (hippocampal subfields). To the best of our knowledge, this is the first study to demonstrate intra-network alternation in the hippocampi of patients with MCI using population-based studies with large sample sizes. We found that hippocampal intra-networks were an independent predictor of MCI. The networks included not only those connecting the ipsilateral hippocampus but also those connecting the bilateral hippocampi. Importantly, these networks predicted MCI independent of hippocampal volume, although hippocampal volume was also a predictor of MCI. Our results suggest that altered networks may reflect different pathologies in MCI from brain atrophy.

The ICA extracted two hippocampal intra-networks (Components 5 and 16) associated with the presence of MCI. Ezama et al. also reported an activity hotspot that extended the hippocampal subfields (CA1, CA3, CA4, subiculum, and dentate gyrus) along the longitudinal axis in healthy participants^8^.

We also found spotty networks (Component 5: right hippocampal head; Component 16: bilateral hippocampal tails). Many studies have demonstrated anatomical and functional differentiation along the long axis of the hippocampus^17^. A previous tract-tracing study of nonhuman primates revealed intra-subfield gradients of connectivity along the anterior–posterior axis of the hippocampus, suggesting different portions of hippocampal subfields may preferentially interact with other brain regions^18^. Dalton et al. showed subfield-to-subfield functional connectivity within different portions along the hippocampal anterior–posterior axis in healthy participants^7^. This evidence supports our results; there are various networks in the hippocampus.

Interestingly, our findings also revealed MCI-related networks in the bilateral hippocampus (Component 16). Although previous findings have highlighted the network along the longitudinal axis as a key contributor to functional organization^17,19^, the underlying function of the bilateral networks remains unclear. In a recent study of healthy adult subjects, networks of the bilateral hippocampi revealed a strong level of functional symmetry during rs-fMRI. This may suggest an underlying function of the bilateral networks in healthy adults. Furthermore, Watanabe et al. revealed bilateral networks in hippocampal structural images using ICA, and these bilateral networks could predict major depressive disorder in controls. Therefore, future research on bilateral hippocampal networks using new analysis methods is needed to reveal their role in cognitive decline.

The MCI-related hippocampal intra-networks in the present study mainly included portions of CA1. Histological studies have shown afferent and efferent projections in the hippocampal subfields to the rest of the brain, showing the subiculum and CA1 are the main sources of extrinsic projections from the hippocampus^9^. Our results may be supported by the previous study using rs-fMRI, which showed that, compared to young participants, older participants had significantly reduced FC between the CA1-subiculum transition region and the entorhinal cortex^7^, suggesting intra-network alternation in the CA1 and subiculum may dovetail with the progression of tau pathology. Although it is commonly linked with AD, tau protein accumulation also occurs during normal aging. The earliest affected regions of tau accumulation during normal aging are regions encompassing the CA1-subiculum transition region^20^.

Our study had several strengths when compared with previous rs-fMRI studies. Previous studies used rs-fMRI and hippocampal subfield segmentation techniques to evaluate hippocampal intra-networks^5,6,8^. However, these studies were limited by their small sample sizes. Our community-based design and large sample size reduced sampling bias and the effects of individual variability and permitted a detailed evaluation of confounding factors. We used 3 T MRI data, although some studies used rs-fMRI with 7 T MRI, which has not been widely used for MR examinations in clinical situations^5,8^. Furthermore, in the present study, all brain MRI data were obtained using the same protocol and MRI scanner. Previous investigations have suggested that volume measurements across platforms (vendor, MRI sequence, and scanner upgrade) introduce difference bias^21,22^.

We evaluated collinearity between the HV/ICV ratio and the 16 independent components, although it was the main aim of our study to evaluate the MCI-related networks in hippocampus, rather than to evaluate the relationship HV/ICV and MCI. The VIF values for the Model 5 indicated no collinearity between the HV/ICV ratio and the 16 independent components. The Pearson’s correlation coefficients also showed a considered indicative of no multicollinearity between them. However, in this study, the HV/ICV was not significant predictors of MCI after adjustment for the 16 intra-networks. Although the reason for this result remains unclear, the hippocampal volume may have somewhat relationship with the hippocampal intra-networks for prediction of MCI pathology because ICA extracted these networks from the hippocampal structural images (hippocampal volume).

A limitation of this study is that fMRI was not performed to confirm the hippocampal intra-networks extracted from the structural MRI. However, a previous study showed a direct association between functional and structural covariance networks using SBM across the entire brain^23^. Furthermore, a correlation between structural and functional networks has been reported in hippocampal intra-networks based on hippocampal subfield segmentation^5^. Although the previous fMRI studies have shown afferent and efferent projections in the hippocampal subfields to the rest of the brain^6,7^, we did not evaluate the relationships with the hippocampal intra-networks and the rest of the brain. The previous 7T MRI study used sMRI with spatial resolution of 0.6 mm × 0.6 mm × 0.6 mm for evaluation of hippocampal subfields^24^. However, our 3D fast-spoiled gradient recalled sequence have a resolution; 1.0 × 1.0 × 1.2 mm, which was similar to that used in previous 3T hippocampal subfields studies^25,26^. Although our sMRI parameter was not high resolution, we believe that our protocol is acceptable to evaluate the hippocampal subfields. Our sample was restricted to relatively well-educated ethnic Japanese individuals, which limits the generalizability of our findings to other racial/ethnic groups, particularly those in less developed countries. Therefore, further studies will be needed to assess altered hippocampal intra-networks in patients with MCI by using 3T MRI datasets from other sources such as Alzheimer's Disease Neuroimaging Initiative (ADNI)^27,28^.

In this population-based study with a large sample size, we extracted the hippocampal intra-networks based on SBM and ICA, which can be estimated using conventional structural imaging with 3T MRI. We identified two MCI-related hippocampal intra-networks. These networks included a novel network connecting the bilateral hippocampi, not only the ipsilateral hippocampus. Moreover, these two networks predicted MCI independently of hippocampal volume, suggesting the altered networks may reflect a different pathology from that of brain atrophy. Therefore, this method provides additional information for understanding cognitive impairment.

## Methods

### Study population and design

This study was conducted in accordance with the ethical guidelines of the Declaration of Helsinki, and use of data from the Iki-Iki Health Promotion Project (Iki-Iki study) was approved by the Ethics Committee of Hirosaki University School of Medicine (authorization number 2019-064-1). Written informed consent was obtained from all participants.

The Iki-Iki Health Promotion Project was established in 2016 as a population-based prospective study of cerebro- and cardiovascular diseases and dementia in an older Japanese population from the Iwaki area of Hirosaki City, western Aomori Prefecture, Japan. In 2016 and 2017, 2390 residents aged > 64 years participated in the screening survey. Of these 2390 residents, 2226 (93.1%) underwent brain MRI. We excluded 50 participants with image distortions (7 with metal artifacts, 13 with excessive motion artifacts, 30 for whom brain volume or structural covariance intra-networks could not be measured accurately for various reasons), and 43 participants without available MRI data (43 without T1WI).

The diagnosis of dementia was made using a two-step diagnostic system. First, an interview survey for the screening of cognitive function was conducted by trained doctors, public health nurses, nurses, and clinical psychologists using the Mini-Mental State Examination (MMSE) as the first screening survey. The subjects who met the following criteria underwent the second screening survey for the suspected cases of cognitive impairment: (1) MMSE ≤ 26 points, (2) score of ≤ 4 of a total possible 6 points on the delayed recall component of the MMSE, (3) failed intersecting pentagon-copying component in the MMSE and/or cube-copying test, and (4) suspected cases based on the manner of speaking and behavior. In the second screening survey, the presence of cognitive impairment (i.e., MCI or dementia) was determined by expert psychiatrists or neurologists based on the physical and neurological examinations.Petersen’s criteria were used for the diagnosis of mild cognitive impairment (MCI)^29^. The diagnosis of AD was based on the criteria of the National Institute of Neurologic and Communicative Diseases and Stroke/Alzheimer Disease and Related Disorders Association^30^. Of the 2133 participants, 218 (9.78%) were diagnosed with MCI and 11 participants were diagnosed with AD. The remaining 1904 (89.3%) patients were CNOA. Eleven participants with AD were also excluded. Thus, 2122 participants (218 with MCI and 1904 CNOA) were enrolled in the present study (Table 1).

### MRI acquisition

All brain MRI data were obtained using the same protocol on a single 3T MRI scanner (Signa EXCITE 3T; GE Healthcare, Wankesha, WI, USA) with an 8-channel brain phased-array coil. Original T1WI were acquired in the steady state using a 3D fast-spoiled gradient recalled sequence with the following parameters: repetition time, 10 ms; echo time, 4.1 ms; inversion time, 700 ms; flip angle, 10; field-of view, 24 cm; section thickness, 1.2 mm; and resolution, 1.0 × 1.0 × 1.2 mm. All images were corrected for distortion due to gradient non-linearity using Grad Warp software^31^ and for intensity inhomogeneity using the “N3” function^32^.

### Image processing

The preprocessing of images was identical to the procedure adopted for classical voxel-based morphometry (VBM) analyses using SPM12 software (Statistical Parametric Mapping 12; Institute of Neurology, London, UK)^33,34^. The structural images in native space were spatially normalized, segmented into grey matter (GM), white matter, and cerebrospinal fluid images, and modulated using the Diffeomorphic Anatomical Registration Through Exponential Lie Algebra (DARTEL) toolbox in SPM12^35^. Ashburner proposed DARTEL as an alternative method of normalization in the SPM package^33^. To preserve the gray and white matter volumes within each voxel, we modulated the images using Jacobian determinants derived from spatial normalization using DARTEL. In contrast to conventional VBM processing, to maintain a high spatial resolution, the voxel size was set at 1.2 mm isotropic voxel size, which is normally converted to a 1.5 mm isotropic voxel size.

Furthermore, the resulting modulated GM images were smoothed using a 3-mm full width at half maximum Gaussian kernel. After the smoothing process, we extracted the hippocampal image, defined by automated anatomical labeling^36^ using the WFU PickAtlas version 3.0.4^37,38^.

To identify structural networks among hippocampal voxels, SBM analysis was performed using the GIFT toolbox (http://icatb.sourceforge.net)^10^. The minimum description length principle was used to estimate the number of independent components. The minimum description length yielded 16 reliable incident components. We performed ICA using a neural network algorithm (Infomax) to minimize the mutual information of the network outputs and identify naturally grouping and maximally independent sources^39^. Independent component analysis was repeated 20 times in ICASSO to ensure the stability of the estimated components.

As a result, we obtained a matrix in which the 2122 rows represented 2122 subjects (1904 CNOA and 218 MCI), and each column indicated a voxel. This matrix was decomposed into two matrices using ICA. The first matrix, called the “mixing matrix,” comprises one subject per row and IC per column. The mixing matrix involved loading coefficients that demonstrated how each structural component contributed to the 2122 participants and thus contained information about the relationship between each participant and each component. In other words, the loading coefficients reflected the contribution of each participant to the specific brain network components. The second matrix was named the “source matrix” and specified the relationship between the ICs and the voxels.

To visualize the independent components, the source matrix was reshaped back to a 3D image, scaled to unit standard deviations (Z maps), and the threshold was set at *Z* > 2.0*.*

ICV, total GMV, total WMV, and bilateral HV were calculated using the 40-brain LPBA40 atlas^40^. We then calculated the GMV/ICV, WMV/ICV, and HV/ICV ratios as indicators of hippocampal atrophy. The analysis was conducted using the CAT12 toolbox (C. Gaser, Structural Brain Mapping Group, Jena University Hospital, Jena, Germany; http://dbm.neuro.uni-jena.de/cat/) implemented within SPM12 software^33,34^.

### Statistical analyses

There were no previous studies in subjects with MCI, which studied the hippocampal intra-network connectivity as measured using SBM; there was no evidence regarding the relationship among the 16 intra-networks. Therefore, for the logistic regression analysis, as explanatory variables, we used all 16 intra-networks extracted by ICA (Model 4 and 5 in Table 2). Furthermore, we investigate the association of cognitive status with each brain volume (GMV/ ICV, WMV/ICV, and HV/ICV ratios) (Model 1, 2, and 3 in Table 2). In the results, the HV/ICV ratio was a significant independent predictor of MCI, but not GMV/ ICV and WMV/ICV. Therefore, to confirm whether the loading coefficients of networks can be independent of hippocampal volume for the prediction of MCI by logistic regression analysis, we used not only the loading coefficients of networks but also the HV/ICV ratio as predictive variables (Model 5 in Table 2).

All statistical analyses were performed using EZR (Saitama Medical Center, Jichi Medical University, Saitama, Japan)^41^. To compare the demographic characteristics between patients with MCI and CNOA, a Mann–Whitney U test was performed to assess differences in age. The chi-squared test was used for sex comparisons. Nominal variables were expressed as percentages and continuous variables as medians (interquartile ranges) or ranges based on distribution.

The logistic regression analysis was used to investigate the association of cognitive status (MCI and CNOA) with hippocampal intra-network connectivity and the HV/ICV ratio. Logistic regression analysis was also used to assess whether hippocampal intra-networks could predict MCI. Bonferroni correction was applied to these results^42^.

Analyses were adjusted for age, sex, education level (less than high school, high school or equivalent, college or graduate, or professional school), and self-reported medical history (hypertension, hyperlipidemia, or diabetes). The diagnosis group (MCI and CNOA) was entered as an independent variable, and all loading coefficients were calculated to indicate hippocampal intra-network connectivity.

### Supplementary Information

Supplementary Table 1.

## Data Availability

The datasets are not publicly available due to privacy protection but are available from the corresponding author on reasonable request.

## Abbreviations

MCI: Mild cognitive impairment
AD: Alzheimer's disease
CNOA: Cognitively normal older adults
SBM: Source-based morphometry
ICA: Independent component analysis
HV: Hippocampal volume
