# Supplementary material for: Altered structural hippocampal intra-networks in a general elderly Japanese population with mild cognitive impairment
Source: Sci Rep. 2023 Aug 16;13:13330. doi: 10.1038/s41598-023-39569-6 (PMC10432547; doi:10.1038/s41598-023-39569-6)
Supplement: Supplementary file 1 — Supplementary Table 1. [file 41598_2023_39569_MOESM1_ESM.docx]

| Supplementary Table S1. Clinical charactaristics of all patients | |  |  |  |
| --- | --- | --- | --- | --- |
|  | Total  (n=2378) | Included participants (n=2122) | Excluded participants  (n=256) | p value |
| Age, median (IQR) | 69 (67-73) | 69 (67-73) | 69.5 (67ｰ74) | 0.086 |
| Sex: male/female | 933/1445 | 823/1299 | 110/146 | 0.199 |
| Diabetes, n (%) | 306(12.9%) | 271 (12.8%) | 35 (13.7%) | 0.398 |
| Hypertension, n (%) | 1106(46.5%) | 980 (46.2%) | 126 (49.2%) | 0.613 |
| Hyperlipidemia, n (%) | 1031(43.4%) | 926 (43.6%) | 105 (41.0%) | 0.635 |
| Education: University/High school/Junior high school | 692/1243/439 | 623/1108/391 | 69/135/52 | 0.747 |
| Cognitive disorders: NOA/MCI | 2130/248 | 1904/218 | 226/30 | 0.45 |
| mild cognitive impairment (MCI), Normal Older Adult (NOA), interquartile range (IQR) | | | | |
